# The microbial diversity following antibiotic treatment of *Clostridioides difficile* infection

**DOI:** 10.1186/s12876-021-01754-0

**Published:** 2021-04-13

**Authors:** Dana Binyamin, Orna Nitzan, Maya Azrad, Zohar Hamo, Omry Koren, Avi Peretz

**Affiliations:** 1 The Azrieli Faculty of Medicine, Bar Ilan University, 1311502 Safed, Israel; 2 Unit of Infectious Diseases, Baruch Padeh Medical Center, 15208 Poriya, Israel; 3 Clinical Microbiology Laboratory, Baruch Padeh Medical Center, Hanna Senesh 818/2, 15208 Poriya, Tiberias, Israel

**Keywords:** *Clostridioides difficile*, Gut microbiome, Infection

## Abstract

**Background:**

*Clostridioides difficile* (*C. difficile*) is a major nosocomial pathogen that infects the human gut and can cause diarrheal disease. A dominant risk factor is antibiotic treatment that disrupts the normal gut microbiota. The aim of the study was to examine the correlation between antibiotic treatment received prior to *C. difficile* infection (CDI) onset and patient gut microbiota.

**Methods:**

Stool samples were collected from patients with CDI, presenting at the Baruch Padeh Medical Center Poriya, Israel. Demographic and clinical information, including previous antibiotic treatments, was collected from patient charts, and CDI severity score was calculated. Bacteria were isolated from stool samples, and gut microbiome was analyzed by sequencing the 16S rRNA gene using the Illumina MiSeq platform and QIIME2.

**Results:**

In total, 84 patients with CDI were enrolled in the study; all had received antibiotics prior to disease onset. Due to comorbidities, 46 patients (55%) had received more than one class of antibiotics. The most common class of antibiotics used was cephalosporins (n = 44 cases). The intestinal microbiota of the patients was not uniform and was mainly dominated by *Proteobacteria*. Differences in intestinal microbiome were influenced by the different combinations of antibiotics that the patients had received (*p* = 0.022)

**Conclusions:**

The number of different antibiotics administered has a major impact on the CDI patients gut microbiome, mainly on bacterial richness.

## Introduction

*Clostridioides difficile* (*C. difficile*) is a Gram-positive, obligate anaerobic bacterium that is a member of the Firmicutes phylum. Its highly resistant spores survive on surfaces for long periods, rendering it highly transmissible from person to person. This occurs mainly in hospitalization facilities, categorizing *C. difficile* infection (CDI) as a nosocomial infection [1]. This bacterium can also colonize the gut asymptomatically, potentially leading to a “silent” onward transmission [2]. Symptomatic infection, also called CDI, is characterized by diarrhea, fever, abdominal pain and an increase in the white blood cell count. The major risk factor for CDI is antibiotic administration, which triggers diarrheal diseases, termed antibiotic-associated diarrhea [3]. Although nearly all antimicrobial classes have been associated with CDI, clindamycin, third generation cephalosporins, fluoroquinolones, and penicillins are most commonly implicated [4].

The gut microbiome plays a central role in CDI. The human body is colonized by a large number of microorganisms, including bacteria, fungi, parasites, and viruses, together termed the human microbiome, whose composition is influenced by several factors, such as diet and host genetics [5]. Opportunistic pathogens are primarily blunted by activation of the immune system [6]. This colonization resistance is altered by antibiotics; bacterial composition, richness, and diversity change (dysbiosis) several days after antibiotic administration, generating a convenient niche for spore germination, proliferation, and toxin production [4]. Indeed, data from human studies have shown that the presence of *C. difficile*, either as a colonizer or as a pathogen, is associated with reduced microbiota diversity. The various antibiotics families may have differential effects on the gut microbiota, thus varying in their impact on predisposition for CDI. The current study examined the changes in the gut microbiota occurring following antibiotic treatment of patients with CDI.

## Methods

### Study population

The study population consisted of adults patients diagnosed with CDI and hospitalized at the Baruch Padeh Medical Center Poriya, Israel. CDI patients mainly suffered from diarrhea. Patients signed a consent form or had a legal guardian sign in their place. Patients with IBD, recurrent CDI, pregnant women and patients suffering from mental illness were not eligible to participate in the study. The study was approved by the Helsinki Committee of the Medical Center, approval No. 0003-15 POR.

### Sample collection and C. difficile detection

*C. difficile* identification in stool samples was performed at the Baruch Padeh Medical Center Clinical Microbiology Laboratory, as part of the routine CDI screening procedures. A polymerase chain reaction (PCR) test was performed using the GeneXpert *C. difficile* PCR assay (Cepheid, Sunnyvale, CA, USA), to identify toxin B, binary toxin, and *tcdC* deletion.

### Severity score calculation

To classify CDI severity, the severity score index (SSI) was calculated according to the "Score indices for *C. difficile* infection severity", [7] which incorporates nine parameters that are associated with increased CDI morbidity and mortality. For each patient, one point is given for each of the following parameters: altered mental status, abdominal pain or distention, 1500 > white blood cell (WBC) > 20,000 cells per cubic meter, hypoalbuminemia (< 2.5 mg/dL albumin, ALB), ascites or colitis (imaging), mean arterial pressure (MAP) < 65 mmHg, tachycardia ≥ 110 beats/min, intensive care unit (ICU) transfer. A score of 0–3 reflects mild disease, 4–6 moderate disease, and ≥ 7 severe disease.

The following demographic and clinical information was collected from the medical records: gender, age, community- versus hospital-acquired CDI, death during hospitalization, and laboratory test (leukocyte count, serum albumin, C-reactive protein (CRP) results).

### Gut microbiome

#### DNA extraction, amplification and sequencing

DNA was extracted from 0.25 ml liquid fecal samples using the Power Soil DNA Isolation Kit (MoBio, Carlsbad, USA), according to the manufacturer's instructions with a 2-min bead-beating step (Biospec) [8]. DNA was stored at − 20 °C until use.

From the extracted DNA, the V4 region of the bacterial 16S rRNA gene was amplified using the 515F and 806R barcoded primers, as per the Earth Microbiome Project protocol [9]. PCR reaction conditions included an initial denaturing step (3 min at 95 °C), 30 cycles of denaturation (10 s at 98 °C), annealing (5 s at 55 °C), and extension (20 s at 72 °C), with a final elongation for 1 min at 72 °C. PCR products were purified using AMPure magnetic beads kit (Beckman Coulter, Florida, USA), [10] and then quantified using the Qubit dsDNA HS Assay kit (Thermo Fisher, Bartlesville, USA) [11]. Samples were pooled to equal concentrations of 50 ng/μl and purified again using 2% E-Gel agarose inserted in an E-Gel PowerBase device (Invitrogen, Carlsbad, USA). Then, DNA fragments were purified from the agarose using NucleoSpin® Gel and PCR Clean-up kit (Macherey–Nagel, Düren, Germany), and sequenced using the Illumina MiSeq platform at the Genomic Center, Faculty of Medicine, Bar-Ilan University, Israel.

#### Analysis

Analysis was performed using QIIME2 [12]. Sequences were demultiplexed by per-sample barcodes and Illumina-sequenced amplicon read errors were corrected by the Divisive Amplicon Denoising Algorithm (DADA2) [13]. A phylogenetic tree was generated and taxonomy was classified using Greengenes reference database at a confidence threshold of 99% [14]. Alpha and beta diversities were calculated based on a feature table containing features observed in at least 40 samples (50%) and on samples containing at least 8000 sequences. Faith’s Phylogenetic Diversity was used to calculated richness, a qualitative measure of community richness that incorporates phylogenetic relationships between taxa [15]. Beta diversity was analyzed using Principal Coordinate Analysis (PCoA) based on weighted UniFrac distance matrices [16].

### Statistical analysis

In Faith’s phylogenetic diversity measure, differences between groups were analyzed using the Kruskal–Wallis (pairwise) test. Weighted UniFrac distances differences were analyzed using the pairwise Permanova test. Statistical significance was defined as *p* < 0.05.

## Results

### Demographic and clinical profiles

Overall, 84 CDI patients, of an average age of 72.42 ± 16.74 years, were enrolled in this study (Table 1). Of the 84 patients, 41 (48.81%) were women. Seventeen patients (20.24%) died during hospitalization. More than half of the patients (55.95%) had a nosocomial CDI. Disease severity was calculated for 81 patients, as the medical information of three ICU patients was inaccessible; 58 (71.6%) patients suffered from mild disease, 20 (24.7%) from moderate disease, and 3 (3.7%) from severe disease. All patients received antibiotics prior to CDI onset due to other illnesses; 38 patients (45.24%) received one class of antibiotics, 32 patients (38.1%) received two classes, 10 patients (11.9%) received three classes, and 4 patients (4.76%) received 4 classes.

**Table 1:** Demographics and baseline characteristics of CDI patients (N = 84)

Parameter	
Age (years), mean ± SD	72.42 ± 16.74
Gender, n (%)	
Male	41 (48.81)
Female	43 (51.19)
Infection acquisition, n (%)	
Nosocomial	47 (55.95)
Community-acquired	37 (44.05)
In-hospital mortality, n (%)	
Alive	67 (79.76)
Dead during hospitalization	17 (20.24)
Disease Severity^a^, n (%)	
Mild	58 (71.6)
Moderate	20 (24.7)
Severe	3 (3.7)
Number of antibiotic classes received before CDI onset, n (%)	
1	38 (45.24)
2	32 (38.1)
3	10 (11.9)
4	4 (4.76)

^a^Disease severity was calculated for 81 patients only

### Antibiotics received before CDI onset

Twelve classes of antibiotics taken before CDI onset were recorded (Table 2). Antibiotics from the cephalosporin class were most widely used (n = 44), followed by penicillin (n = 32), which was often administered with other antibiotics (n = 24). Sulfa antibiotics were used in only 2 patients and tetracyclines in one patient. In 18 cases, one antibiotic from the cephalosporin class was sufficient to trigger disease, but CDI was often diagnosed after treatment with cephalosporin in combination with other antibiotics (n = 26).

**Table 2:** Antibiotics classes received by CDI patients

Antibiotic class	1 class of antibiotic n = 38	2 classes of antibiotic n = 32	3 classes of antibiotic n = 10	4 classes of antibiotic n = 4	Overall
Aminoglycoside	2	6	0	3	11
Carbapenem	2	4	3	0	9
Cephalosporin	18	17	7	2	44
Chloramphenicol	2	3	0	1	6
Glycopeptide	0	2	4	2	8
Lincosamides	4	1	1	1	7
Macrolide	1	1	4	0	6
Penicillin	8	16	6	2	32
Quinolone	1	6	2	2	11
Tetracycline	0	1	0	1	2
Trim/sulfa	0	1	0	0	1
Other	0	6	3	2	11

Some patients received more than one antibiotic

### Microbiome analysis

**Fig. 1:**
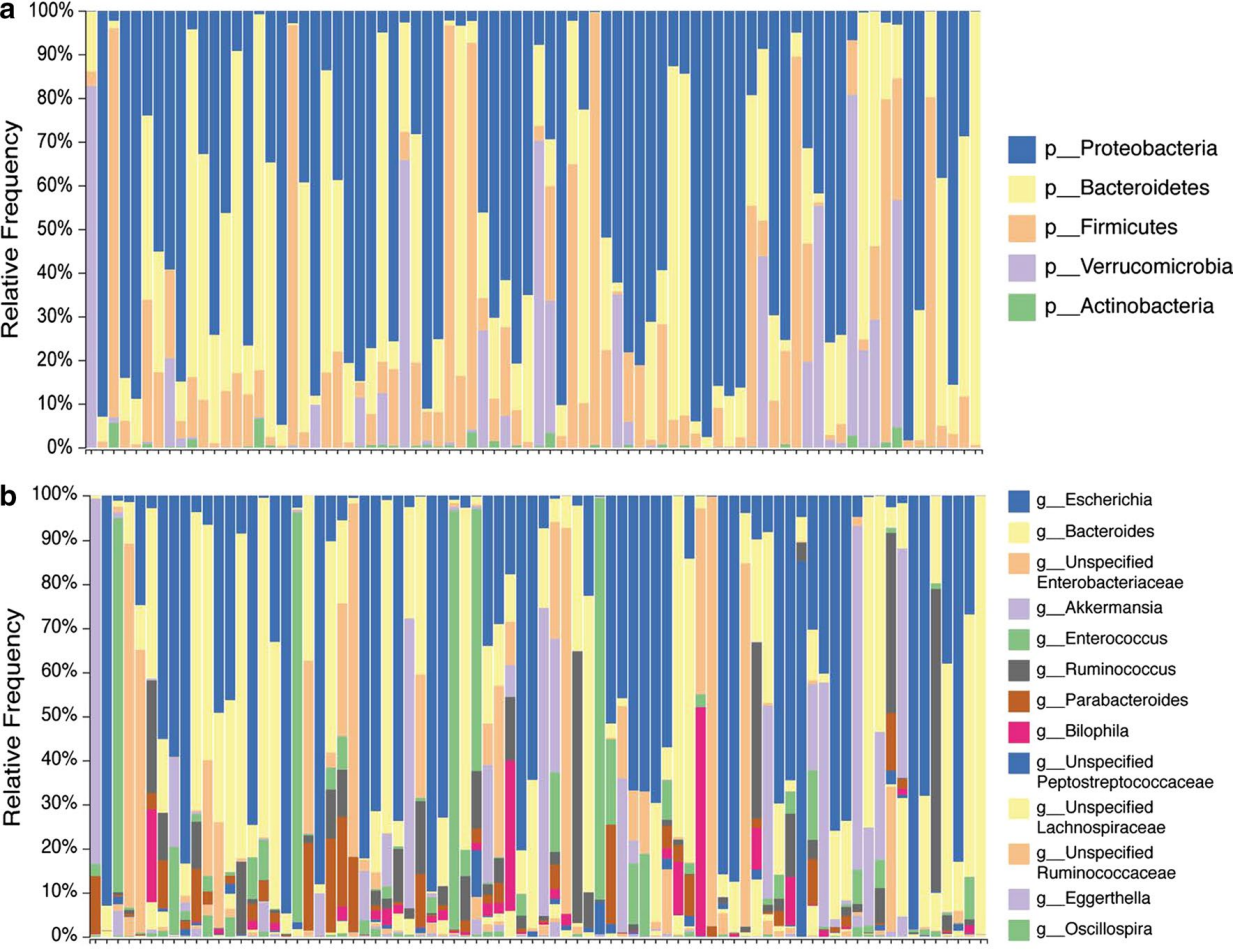
Microbial community structure of CDI patients. The gut microbiota composition of 67 CDI patients. Taxonomy plot at **a** phylum and **b** family levels

Microbiome analysis was performed on 67 out of 84 CDI patient samples (79.76%); 17 samples (20.24%) did not pass quality control. *Proteobacteria* were the dominant phylum in 50% of samples, followed by Bacteroidetes (20%) (Fig. 1) and *Firmicutes* and *Verrucomicrobia* phyla (12%) (Fig. 1a). At the family level, Enterobacteriaceae and Bacteroidaceae each dominated 20% of the patient samples and Verrucomicrobiaceae dominated 11% of the samples. Other families were present in smaller percentages (Fig. 1b).

**Fig. 2:**
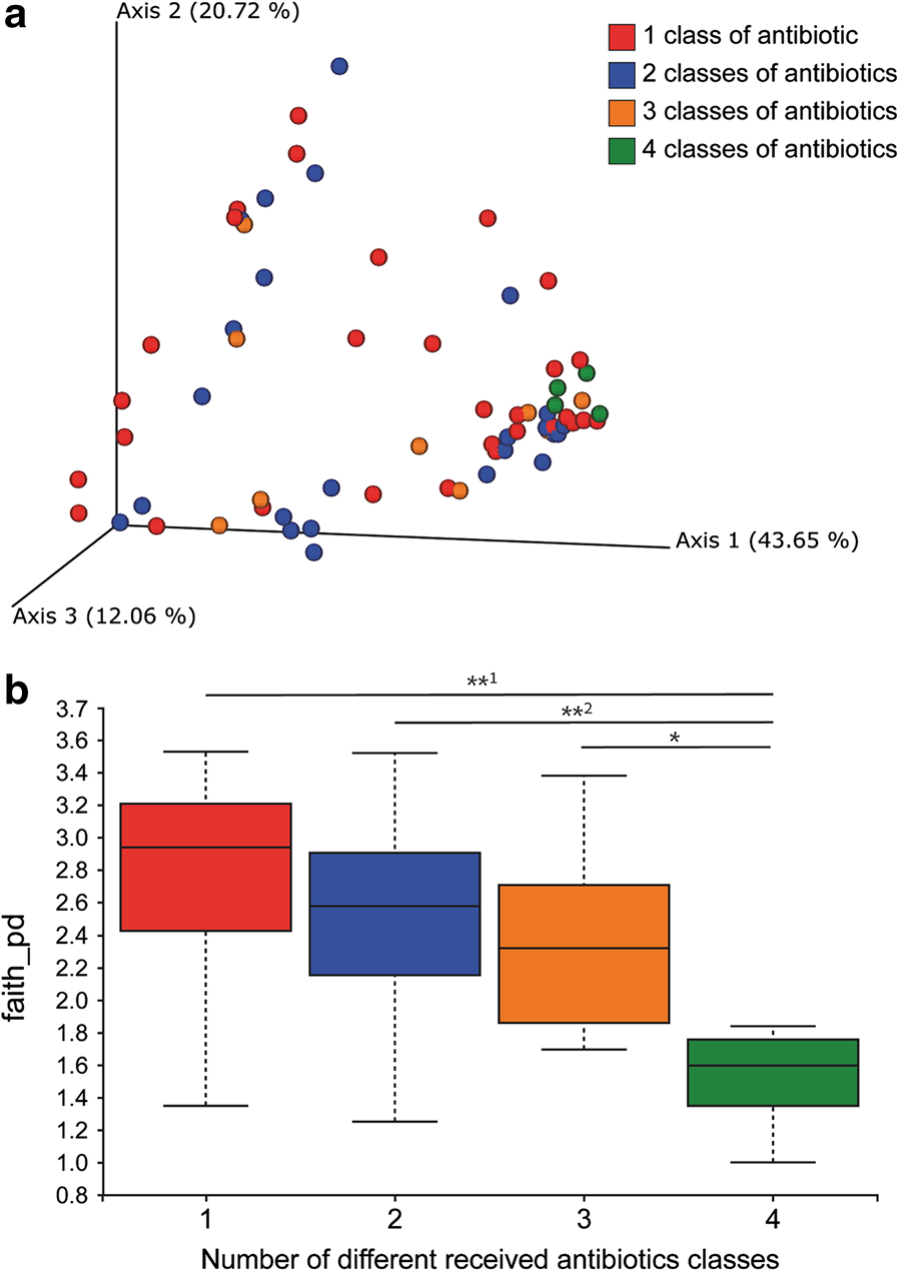
Microbial community diversity between and within CDI patients, by combinations of antibiotics received prior to CDI onset. CDI patients were divided to four groups according to the number of classes of antibiotics received prior to onset of infection: 1 (n = 30), 2 (n = 24), 3 (n = 9), and 4 (n = 4) classes of antibiotics. **a** Beta diversity using weighted UniFrac. **b** Alpha diversity using Faith’s Phylogenetic Diversity (*p = 0.022)

Calculation of beta-diversity in samples from CDI patients who had received different combinations of antibiotics (Fig. 2a) showed that only patients who had received four classes of antibiotics clustered significantly distant from the other groups (*p* < 0.05). Bacterial richness (alpha-diversity) negatively correlated with the number of antibiotics received (Fig. 2b). Bacterial richness among CDI patients who had received four classes of antibiotics was the lowest and was significantly different from the other groups (*p = 0.03, **^1^*p* = 0.007, **^2^*p* = 0.005).

## Discussion

The current study examined the correlation between gut bacterial composition of CDI patients and antibiotic treatment received prior to infection onset. The epidemiological data of the study population was in correlation with the known characteristics of CDI patients, i.e., older age and high mortality rate. Additionally, the numbers of nosocomial- and community-acquired cases were similar to earlier reports [17]. Recently, the prevalence of community-acquired infections has increased due to elevated use of antibiotics that were previously only administered in hospitals via intravenous infusion [17].

Most patients were diagnosed with mild disease, while only a few were diagnosed with severe disease. These results point to an increase in the prevalence of moderate-severe CDI compared to a study conducted in 2016 in northern Israel which found that most patients had mild disease, a few had moderate disease, and none were diagnosed with a severe disease [18]. This increase in disease severity can be attributed to an increase in antibiotic resistance or emergence of more virulent strains [1].

The majority of patients received one or two classes of antibiotics prior to CDI onset, corresponding with previous reports demonstrating that one type of antibiotic is sufficient to induce CDI [4]. Cephalosporins and penicillins were the most commonly used antibiotics, two drugs which have previously been shown to significantly increase the risk of CDI as compared to other antibiotics [4, 19, 20]. Fluoroquinolone and clindamycin have also been highly correlated with CDI development, yet, in our study, only a small percentage of patients received these antibiotics.

Examination of intestinal bacterial populations of CDI patients and their correlation with previous antibiotic treatment, showed that there was no phylum- and family-level composition common to all CDI patients, as has been described in other studies [21–23]. In their study comparing the gut microbiome profile of CDI versus non-CDI patients, Manges et al. found an increase in *Firmicutes*, *Proteobacteria* and Actinobacteria phyla, as well as a decrease in *Bacteroidetes* [24]. Antharam et al., surveying the distal gut microbiota of individuals with CDI, found that these patients had significantly less diverse communities, particularly a less diverse *Firmicutes* population than patients with non-CD diarrhea or healthy controls [21]. In addition, there was depletion of gut commensals such as the Ruminococcaceae and Lachnospiraceae families and butyrate-producing anaerobic fermenters. One of the genera discovered in this study is *Akkermansia*, a human intestinal mucin-degrading bacterium which may contribute to *C. difficile* establishment by damaging the essential mucus layer preventing against gut pathogens [24]. This lack of uniformity can be explained by the various factors affecting the intestinal bacteria, such as nutrition, [25] although we tried to control for these factors during data analysis. More specifically, several parameters (such as age, gender, and disease severity) were tested, yet none had significant effects on bacterial population. In contrast, we found that the antibiotic combination administered to CDI patients before disease onset correlated with the intestinal microbiota. Patients who had received four classes of antibiotics had more similar microbiomes. In addition, an inverse correlation between bacterial richness and the number of antibiotics received was noted, with significant differences between patients who received four classes of antibiotics versus those who received one or two classes of antibiotics. These findings can likely be ascribed to the broader range of bacterial species targeted by multi-class antibiotic treatment regimens, which subsequently leads to reduced microbiota richness. A limitation of this study was the lack of a comparison to the gut microbiome of healthy individuals, due to the difficulty in finding healthy elderly controls. Such a comparison may have provided insights into the importance of the gut microbiota's capability of providing colonization resistance against *C. difficile*.

## Conclusions

No uniform microbiome profile was observed among the tested CDI patients. Yet, the gut microbiome of patients who had received four different antibiotics classes, demonstrated significantly lower richness and diversity compared to patients who received fewer than four different antibiotics classes.

## Data Availability

The datasets used and/or analyzed during the current study are available from the corresponding author on reasonable request.

## Abbreviations

*C. difficile*: *Clostridioides difficile*
CDI: *C. difficile* infection
SSI: Severity score index
PCR: Polymerase chain reaction
